# Roasting Time Shapes Quality Attributes, Aroma Formation and Oxidative Lipid Remodeling in Almond Oil

**DOI:** 10.3390/foods15162842

**Published:** 2026-08-14

**Authors:** Shengjie Ding, Liangli Chen, Batuer Guliziba, Yang Zhao, Xingxing Deng, Songyi Lin, Zhiqiang Lu

**Affiliations:** 1 College of Food Science and Pharmaceutics, Xinjiang Agricultural University, Urumqi 830002, China; 18703003661@163.com (S.D.); 17726781796@163.com (L.C.); 13239085119@163.com (B.G.); 18889202331@163.com (Y.Z.); m13029617289@163.com (X.D.); 2 Xinjiang Tacheng Food and Drug Inspection Institute, Tacheng 834700, China; 3 National Engineering Research Center of Seafood, School of Food Science and Technology, Dalian Polytechnic University, Dalian 116034, China; linsongyi730@163.com

**Keywords:** almond oil, roasting time, GC-IMS, machine learning, lipidomic, volatile compounds, oxidative stability

## Abstract

Roasting time strongly influences almond oil yield, flavor and oxidative stability. This study investigated almond oils obtained by pressing almond kernels roasted at 130 °C for 0, 5, 10 and 20 min, corresponding to cold-aroma, light-aroma, strong-aroma and sauce-aroma groups, respectively, by combining physicochemical analysis, fatty acid quantification, gas chromatography–ion mobility spectrometry (GC-IMS), machine learning and lipidomics. Increasing roasting time enhanced oil yield from 45.81% to 50.34%, with no further significant increase after 10 min. Stronger roasting moderately reduced the free radical level. In contrast, acid value, peroxide value and thiobarbituric acid reactive substances (TBARS) increased from 0.28 to 0.38 mg KOH/g oil, 1.20 to 1.46 meq O_2_/kg oil and 0.38 to 0.46 mg malondialdehyde/kg oil, respectively, indicating limited oxidative deterioration. Fatty acid analysis showed that oleic acid remained relatively stable at 644.37–647.03 mg/g oil, whereas linoleic acid decreased significantly from 150.42 to 140.19 mg/g oil. GC-IMS detected 59 volatile signals and clearly separated the four processing groups, indicating substantial roasting-induced changes in volatile fingerprints. A positive–unlabeled (PU) learning model used 27 literature-supported aroma-active compounds as the positive class and 32 compounds without confirmed aroma-activity evidence as the unlabeled class. The model showed good cross-validated discrimination, with a mean area under the receiver operating characteristic curve (ROC-AUC) of 0.866 and a mean area under the precision–recall curve (PR-AUC) of 0.756, and prioritized eight unlabeled compounds, mainly alcohols and methional, as candidate aroma-active compounds requiring further sensory validation. Lipidomic analysis showed that accelerated oxidation caused broad lipid remodeling, with 327 differential lipids between cold-aroma oxidized and unoxidized oils, mainly involving glycerophospholipids, glycerol lipids, sphingolipids and fatty acyls. Overall, increasing roasting time improved oil recovery and promoted aroma differentiation while causing only limited initial oxidative deterioration, providing a theoretical basis for optimizing roasting conditions in industrial processing.

## 1. Introduction

Almond (*Prunus dulcis*) is an important tree nut crop rich in lipids, proteins, dietary fiber and minerals, and contains minor bioactive compounds, including tocopherols, phytosterols and phenolic compounds [1]. Almond oil is characterized by a high proportion of unsaturated fatty acids, particularly oleic acid (approximately 60–75% of total fatty acids) and linoleic acid (approximately 15–30% of total fatty acids), and is valued for its nutritional quality and potential application in edible oil and functional food products [2,3,4]. The quality of virgin almond oil is affected by cultivar, raw material characteristics and extraction conditions, which influence oil yield, fatty acid composition, minor components and sensory properties [2,3,5]. Therefore, improving oil recovery while maintaining desirable oxidative stability and flavor quality is an important objective in almond oil processing.

Roasting is a common pretreatment for nut oil production because it can promote oil release and generate characteristic roasted aroma. Heat treatment can disrupt cellular structures, weaken the interaction between oil bodies and surrounding matrices, and facilitate oil migration, thereby improving extraction efficiency [5,6]. Meanwhile, roasting induces the Maillard reaction, Strecker degradation, caramelization and lipid oxidation, which jointly contribute to color development and volatile aroma formation [7,8,9]. Previous studies have shown that roasting temperature and duration affect almond color, phenolic composition, antioxidant capacity, Maillard reaction degree and volatile profiles [7,8]. Volatile compounds such as pyrazines, aldehydes, furans and related compounds are important contributors to nutty, roasted, fatty and caramel-like notes in roasted almonds [9,10]. However, overly intense roasting can hasten oxidative deterioration by promoting hydroperoxide formation and secondary oxidation products, thereby compromising oil quality during processing or storage [11,12,13]. Thus, an appropriate roasting time should balance oil yield, flavor development and oxidative stability.

Although the effects of roasting on almond kernels and some quality indices of almond oil have been reported, several limitations remain. First, most studies have focused on individual aspects, such as color, antioxidant activity, fatty acid composition or volatile compounds, rather than integrating oil yield, physicochemical quality, flavor fingerprints and oxidative lipid changes. Second, conventional oxidation indicators, including acid value, peroxide value and thiobarbituric acid reactive substances (TBARS), provide only overall information and cannot reveal which lipid classes or molecules are preferentially altered during oxidation. Third, the key aroma compounds associated with different roasting times are often discussed based on peak abundance or known odor characteristics, but data-driven screening approaches are still limited. Therefore, a more integrated analytical strategy is needed to clarify how roasting time shapes almond oil quality, aroma formation and oxidation-related lipid remodeling.

Gas chromatography–ion mobility spectrometry (GC-IMS) enables rapid volatile fingerprinting and is widely applied for non-targeted profiling of food aroma [14]. Combined with machine learning, GC-IMS data can be further used to prioritize candidate aroma-active compounds from complex volatile datasets. Positive–unlabeled (PU) learning is a machine-learning framework used when labeled positive examples are available, whereas the remaining observations are unlabeled rather than confirmed negative examples [15]. In the present study, the literature-supported aroma-active compounds were assigned to the positive class, while the other GC-IMS-detected volatile compounds were treated as unlabeled because insufficient evidence of aroma activity does not indicate that they are aroma-inactive. The PU model was therefore used to rank candidate aroma-active compounds within the unlabeled set rather than to directly confirm their sensory contributions. Random Forest feature importance analysis can further identify chromatographic and abundance variables contributing to model prediction [16]. In parallel, lipidomics enables molecular-level characterization of lipid remodeling, including changes in triacylglycerols, oxidized triacylglycerols, glycerophospholipids, sphingolipids and fatty acyls [17,18,19,20]. These approaches can complement conventional quality analysis and provide deeper insight into roasting- and oxidation-induced changes in almond oil.

This study examined how roasting time shaped quality development, volatile aroma profiles and oxidative lipid changes in almond oil pressed from kernels roasted for 0, 5, 10 and 20 min, representing the cold-aroma, light-aroma, strong-aroma and sauce-aroma groups, respectively. To achieve this, physicochemical analysis (oil yield, color, apparent viscosity, antioxidant-related capacity, oxidation-related indices), fatty acid quantification, GC-IMS, machine learning and lipidomics were integrated. This study is expected to provide a comprehensive understanding of roasting-induced changes in almond oil and offer a theoretical basis for optimizing roasting conditions to balance oil yield, flavor development and oxidative quality.

## 2. Materials and Methods

### 2.1. Materials and Chemicals

Raw almond kernels (*Prunus dulcis*) were obtained from Beiyuanchun Market (Urumqi, Xinjiang, China). Broken, moldy or otherwise visibly damaged kernels were removed by manual sorting, and the remaining kernels were kept in a dry, cool environment before roasting and oil extraction. Unless otherwise specified, all chemicals were of analytical grade. Chromatographic solvents used for GC and lipidomic analyses were of chromatographic or LC–MS grade. The manufacturers and countries of the assay kits, analytical standards, internal standards and major reagents are specified in the corresponding analytical subsections.

### 2.2. Preparation of Almond Oils with Different Roasting Times

Almond kernels were roasted at 130 °C using an electric oven (PT3515, Midea, Foshan, China). Unroasted kernels served as the 0 min control, while the other kernels were roasted for 5, 10 or 20 min. According to the roasting time, the resulting oils were designated as cold-aroma (0 min), light-aroma (5 min), strong-aroma (10 min) and sauce-aroma (20 min), respectively. These terms were used as processing-based group designations rather than categories established through formal sensory evaluation. Immediately after treatment, the kernels were pressed using an oil press (WOP-KPO202, Westinghouse, Cranberry Township, PA, USA). The resulting crude oils were transferred into sealed brown glass bottles and stored at low temperature until analysis.

### 2.3. Determination of Moisture Content and Oil Yield

The moisture contents of almond kernels subjected to different roasting times were determined in triplicate using oven drying (DHG-9030A, Shanghai Yiheng, Shanghai, China) at 105 °C until constant mass and were expressed on a wet-weight basis. After each roasting treatment, the actual kernel mass immediately before pressing and the mass of extracted oil were recorded. Oil yield was calculated as the ratio of the mass of extracted oil to the mass of almond kernels used for pressing. To account for differences in kernel moisture among the roasting treatments, oil yield was additionally normalized to kernel dry matter according to the following equation: Dry-basis oil yield (%) = extracted oil mass/[kernel mass before pressing × (1 − moisture content/100)] × 100.

### 2.4. Determination of Physicochemical Properties

#### 2.4.1. Apparent Viscosity Measurement

The apparent viscosity of almond oil was determined using a rheometer (MCR51, Anton Paar, Graz, Austria) equipped with a concentric cylinder geometry according to previously reported methods with minor modifications [21]. An appropriate amount of almond oil was loaded into the measuring cup, and the apparent viscosity was measured at room temperature. The shear rate was set from 0.01 to 10 s^−1^, and the viscosity changes in almond oils with different roasting times were recorded.

#### 2.4.2. Color Measurement

The color of almond oil was measured using a colorimeter (NR60CP, 3nh Technology Co., Ltd., Shenzhen, China). Before measurement, the instrument was calibrated according to the manufacturer’s instructions. Almond oil was transferred into a transparent sample container, and the color parameters were recorded as L* (lightness), a* (redness/greenness) and b* (yellowness/blueness). The total color difference (ΔE) was calculated using the cold-aroma almond oil as the reference, and the hue angle (H°) was calculated to further describe color changes among oils with different roasting times.

#### 2.4.3. Determination of Antioxidant-Related Capacity

The 2,2-diphenyl-1-picrylhydrazyl (DPPH) (Cat: BC4750), 2,2′-azino-bis(3-ethylbenzothiazoline-6-sulfonic acid) (ABTS) (Cat: BC4770) and ferric reducing antioxidant power (FRAP) (Cat: BC1310) assay kits (Solarbio Biotechnology Co., Ltd., Beijing, China) were used to assess the antioxidant-related capacity of almond oil. Before analysis, the oil samples were dissolved in 80% ethanol and centrifuged at 12,000 rpm for 5 min at 4 °C, and the resulting supernatants were used for the assays. For the FRAP assay, 5 μL of sample extract was mixed with 180 μL of freshly prepared working solution, incubated at 37 °C for 3–5 min and measured at 593 nm. For the ABTS assay, 10 μL of sample extract was mixed with 20 μL of peroxidase working solution and 170 μL of ABTS working solution, incubated at room temperature for 6 min and measured at 405 nm. The DPPH assay was performed using a freshly prepared ethanol-based DPPH working solution, and the absorbance was measured at 515 nm according to the kit instructions. Trolox was used as the standard, and the results were calculated from the corresponding Trolox calibration curves. The antioxidant capacity was expressed as μmol Trolox equivalents per gram of almond oil (μmol TE/g oil).

#### 2.4.4. Determination of Oxidation-Related Quality Indices

The oxidation-related quality indices of almond oil, including acid value, peroxide value and thiobarbituric acid reactive substances (TBARS), were determined to evaluate hydrolytic rancidity and lipid oxidation. Acid value and peroxide value were determined according to previously reported titrimetric methods with minor modifications [22,23]. Briefly, acid value was measured by titrating almond oil dissolved in a neutralized ethanol–ether mixed solvent with potassium hydroxide solution and expressed as mg KOH/g oil. Peroxide value was determined by iodometric titration after reacting almond oil with saturated potassium iodide in an acetic acid–chloroform mixed solvent and expressed as meq O_2_/kg oil. TBARS was quantified according to a previously reported direct thiobarbituric acid colorimetric method with minor modifications [24]. Briefly, approximately 0.20 g of almond oil was accurately weighed into a 25 mL volumetric flask, dissolved in 1-butanol, and diluted to volume with the same solvent. A 5 mL aliquot of the oil solution was mixed with 5 mL of 0.2% (*w*/*v*) thiobarbituric acid solution prepared in 1-butanol. The mixture was heated in a water bath at 95 °C for 2 h and then cooled to room temperature. The absorbance was measured at 532 nm against a reagent blank using a UV–visible spectrophotometer (T700, Beijing PERSEE, Beijing, China). TBARS values were calculated from a malondialdehyde calibration curve and expressed as milligrams of malondialdehyde (MDA) per kilogram of oil.

### 2.5. Fatty Acid Composition Analysis

Fatty acid composition was determined from fatty acid methyl esters using a suitably modified published procedure [25]. In brief, almond oil was mixed with an internal standard solution and saponified with NaOH–methanol solution. The mixture was heated at 80 °C for 5 min, followed by methylation with 14% BF3–methanol solution for 2 min. After cooling to room temperature, fatty acid methyl esters were extracted with n-hexane and subjected to gas chromatography analysis. Fatty acid methyl esters were separated using an Agilent 7890B gas chromatograph equipped with a flame ionization detector (GC-FID; Agilent Technologies, Santa Clara, CA, USA) and a 100 m × 0.25 mm i.d. × 0.20 μm film-thickness capillary column. The injector and detector temperatures were set at 220 °C and 260 °C, respectively. Nitrogen was used as the carrier gas. Peak identities were assigned by comparing the retention time of the sample peaks with those of a commercial 37-component fatty acid methyl ester reference mixture (Supelco 37 Component FAME Mix, Sigma-Aldrich, St. Louis, MO, USA). The reference mixture covered saturated, monounsaturated and polyunsaturated fatty acid methyl esters ranging from C4 to C24, including cis/trans isomers and n − 3 and n − 6 fatty acids. Quantification was performed using calibration curves prepared from the fatty acid methyl ester standards. The peak-area ratio of each fatty acid methyl ester to the internal standard was related to the corresponding concentration ratio. The calculated contents were corrected for the final volume and dilution factor and expressed as mg/g oil.

### 2.6. GC-IMS Analysis of Volatile Compounds

Volatile compounds in almond oil were analyzed using a gas chromatography–ion mobility spectrometry system (GC-IMS; FlavourSpec^®^, G.A.S., Dortmund, Germany) coupled with a CTC-PAL 3 static headspace autosampler (CTC Analytics AG, Zwingen, Switzerland), following a previously reported method with minor modifications [26]. Separation was performed on an MXT-WAX capillary column (15 m × 0.53 mm i.d., 1 μm film thickness; Restek, Bellefonte, PA, USA). For headspace analysis, 400 μL of almond oil was placed in a 20 mL headspace vial, diluted to a final volume of 2 mL with ultrapure water, and supplemented with 10 μL of 2-methyl-3-heptanone solution (0.01 μg/mL) as the internal standard. The sealed vial was equilibrated at 60 °C for 20 min with agitation at 500 rpm. After incubation, 500 μL of headspace gas was automatically injected into the GC-IMS system, and the injector temperature was maintained at 85 °C. The total analysis time was 35 min. The GC column temperature was maintained at 60 °C, and nitrogen served as both the carrier and drift gas. The drift tube was maintained at 45 °C. During analysis, the drift gas flow remained at 150 mL/min, whereas the carrier-gas flow increased from 2 mL/min at 0 min to 10 mL/min at 10 min, 100 mL/min at 20 min and 150 mL/min at 30 min, then stayed at 150 mL/min until completion. The stepped carrier-gas program was used to maintain adequate separation of early-eluting volatile compounds at relatively low flow rates while accelerating the elution of later-eluting compounds and shortening the total analysis time. The same flow program was applied to all samples. The GC-IMS data were processed using VOCal software (version 0.4.03; G.A.S., Dortmund, Germany). Volatile compounds were tentatively identified by matching their experimental retention indices and drift time with those in the integrated GC × IMS Library of VOCal software. Because carrier-gas flow affects chromatographic retention behavior, the programmed increase may influence the measured retention time and the confidence of library matching but does not alter the reference information stored in the library. Therefore, compound identification was based on the combined agreement of retention index and drift time rather than retention time alone. Peak intensities were subsequently used for fingerprinting and multivariate analysis. Before principal component analysis (PCA), the peak-intensity matrix containing 59 volatile-signal variables was mean-centered and scaled to unit variance using Z-score standardization. PCA was performed in Python 3.10.0 using the PCA module in scikit-learn version 1.5.2.

### 2.7. Machine-Learning-Assisted Screening of Candidate Key Aroma Compounds

Positive aroma compounds were compiled from 20 published datasets covering nut oils (almond, peanut, walnut, camellia seed and sesame oils), truffle and frying oils. Detailed information on the sample matrix, analytical method, aroma-activity criterion, the literature source and compounds obtained from each dataset is provided in Table S1. Compounds with an odor activity value (OAV) or relative OAV greater than 1 were defined as the literature-supported aroma-active compounds and assigned as the positive class (P). To align the literature data with the almond oil GC-IMS dataset, compound names were standardized by synonym unification, removal of GC-IMS ion-form suffixes, normalization of Greek letters and deletion of non-alphanumeric characters. After matching, 27 of the 59 detected volatile compounds were labeled as positive, while the remaining 32 compounds were treated as unlabeled (U). This setting was used because reliable negative labels cannot be experimentally verified without exhaustive sensory omission or recombination tests.

For each compound, GC-IMS-derived physicochemical, chromatographic and abundance descriptors were compiled, including molecular weight (MW), retention index (RI), retention time (Rt), drift time (Dt), group-wise mean peak intensities and standard deviations, total peak intensity and coefficient of variation (CV). Chemical-class descriptors were encoded using one-hot variables. Numerical features were standardized and clipped to [−3.5, 3.5] to reduce the influence of extreme values. A BaggingPuClassifier was implemented using a Random Forest base learner (800 trees, max_depth = 6, class_weight = balanced_subsample) with 100 bagging estimators and 85% bootstrap sampling. Model performance was assessed using 5-fold stratified cross-validation, with standardization fitted separately within each training fold to prevent data leakage. The area under the receiver operating characteristic curve (ROC-AUC), area under the precision–recall curve (PR-AUC), precision, recall and F1-score were recorded for each fold. After cross-validation, the final model was trained on all 59 compounds, and each compound was assigned a PU_score. Candidate key aroma compounds were defined within the unlabeled set by applying the 75th percentile of unlabeled PU_scores and a minimum cutoff of 0.30; for this dataset, this rule yielded a final threshold of 0.374. Feature importance was assessed from the impurity-based ranking of the Random Forest base learners [16]. All machine-learning analyses were implemented in Python 3.10.0 using scikit-learn 1.5.2 for the RandomForestClassifier and pulearn 0.0.11 for the BaggingPuClassifier. The fold-level performance metrics, complete signal-level descriptors, PU labels, PU scores, prediction categories, and the full 21-feature ranking with standard deviations across the 100 bagging estimators are provided in Table S2.

### 2.8. Accelerated Oven Oxidation Treatment and Lipidomic Analysis

Accelerated oxidation was performed to evaluate the oxidative lipid changes in almond oil. Three lipidomic groups were analyzed: unoxidized cold-aroma oil, oxidized cold-aroma oil and oxidized strong-aroma oil. Cold-aroma oil, obtained from unroasted kernels, was selected as the baseline treatment. Strong-aroma oil was selected as a representative roasted treatment because it exhibited a substantially increased oil yield and clear roasting-related quality changes, whereas further roasting to the sauce-aroma stage produced little additional increase in oil yield. This experimental design enabled comparison of cold-aroma oil before and after accelerated oxidation and comparison of cold-aroma and strong-aroma oils under identical oxidation conditions. For accelerated oxidation, the cold-aroma and strong-aroma oils were placed in glass containers and incubated in an oven (DHG-9030A, Shanghai Yiheng, China) at 60 °C for 15 days.

Untargeted lipidomic analysis was performed using an ultra-high-performance liquid chromatography–high-resolution tandem mass spectrometry (UHPLC–MS/MS) platform. Before extraction, almond oil samples were thoroughly vortexed. Each oil sample (20 μL) was combined with 200 μL of butanol–methanol solution (1:1, *v*/*v*) containing 10 mM ammonium formate, vortexed for 30 s, sonicated for 1 h at room temperature and centrifuged at 13,000× *g* for 10 min. The supernatant was then collected for UHPLC–MS/MS analysis. Quality control samples were generated by combining equal aliquots from all samples. Lipids were separated on a Shimadzu LC-30 UHPLC system (Shimadzu Corporation, Kyoto, Japan) equipped with a Hypersil GOLD™ C18 column (100 × 2.1 mm, 3 μm; Thermo Fisher Scientific, Waltham, MA, USA; catalog No. 25003-102130). Mass spectra were acquired using a Q Exactive Plus high-resolution mass spectrometer (Thermo Fisher Scientific, USA) operated in both positive- and negative-ion modes. Raw data were processed using MS-DIAL software (v4.70) for peak detection, alignment and lipid annotation [27]. Before principal component analysis (PCA), the lipid-abundance matrix was mean-centered and scaled to unit variance to reduce the influence of differences in signal magnitude among lipid species. PCA was performed using Python 3.10.0 with the PCA module in scikit-learn version 1.5.2. Lipid nomenclature followed the Lipid Metabolites and Pathways Strategy (LIPID MAPS) shorthand notation for mass spectrometry (MS)-derived lipid structures [28]. Differential lipids were screened using an absolute log_2_-transformed fold change (|log_2_FC|) greater than 1.5 and a false discovery rate (FDR) below 0.05.

### 2.9. Statistical Analysis

Unless otherwise specified, experiments were conducted in triplicate and data are presented as mean ± standard deviation. Statistical analyses were performed using IBM SPSS Statistics version 22 (IBM Corp., Armonk, NY, USA). Differences among almond oils with different roasting times were evaluated by one-way analysis of variance (ANOVA), followed by Duncan’s multiple range test. A value of *p* < 0.05 was considered statistically significant.

## 3. Results and Discussion

### 3.1. Effects of Roasting Time on Physicochemical Properties of Almond Oil

#### 3.1.1. Oil Yield

**Figure 1:**
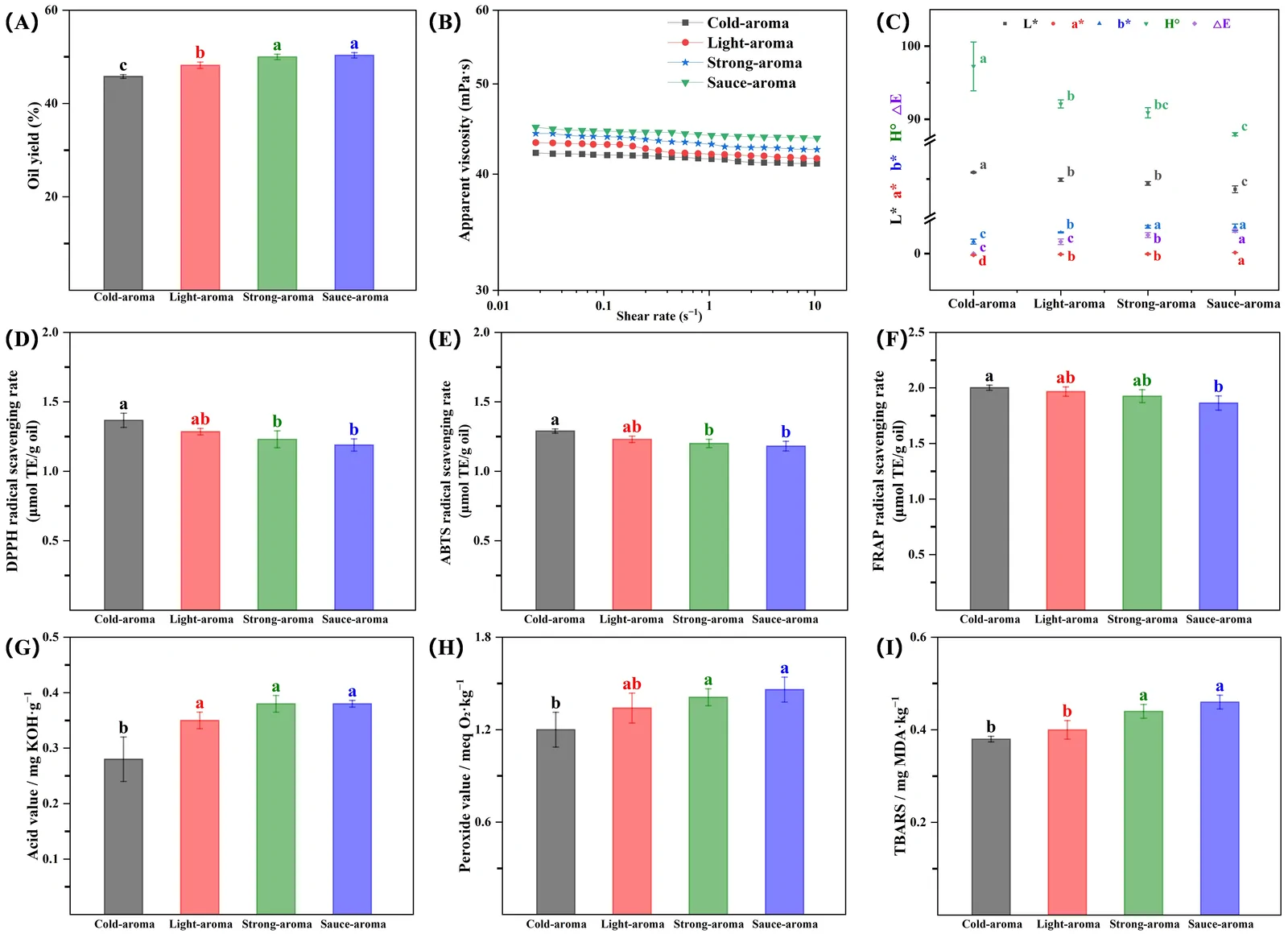
Effects of roasting time on the physicochemical properties of almond oil: (**A**) oil yield expressed on an original-mass basis; (**B**) apparent viscosity as a function of shear rate; (**C**) color parameters, including L*, a*, b*, hue angle (H°), and total color difference (ΔE); (**D**) DPPH radical-scavenging capacity; (**E**) ABTS radical-scavenging capacity; (**F**) ferric reducing antioxidant power (FRAP); (**G**) acid value; (**H**) peroxide value; and (**I**) thiobarbituric acid reactive substances (TBARS). Values are expressed as the mean ± standard deviation of three independent replicates. Different lowercase letters indicate significant differences among roasting treatments for the same parameter (*p* < 0.05). Cold-aroma, light-aroma, strong-aroma and sauce-aroma refer to almond oils obtained from kernels roasted at 130 °C for 0, 5, 10 and 20 min, respectively.

Roasting time significantly influenced almond kernel oil yield (Figure 1A). The cold-aroma group showed the lowest oil yield, with a value of 45.81 ± 0.40%, while the light-aroma, strong-aroma and sauce-aroma groups showed higher values of 48.20 ± 0.68%, 50.00 ± 0.57% and 50.34 ± 0.57%, respectively. Compared with the cold-aroma group, the oil yield increased by 5.22%, 9.15% and 9.90% in the light-aroma, strong-aroma and sauce-aroma groups, respectively. Significant differences were observed among the cold-aroma, light-aroma and strong-aroma groups (*p* < 0.05), whereas no significant difference was found between the strong-aroma and sauce-aroma groups. The moisture contents of the almond kernels were 3.04 ± 0.05%, 2.84 ± 0.03%, 2.75 ± 0.05% and 2.70 ± 0.02% in the cold-aroma, light-aroma, strong-aroma and sauce-aroma groups, respectively, indicating a gradual reduction in moisture with increasing roasting time. To account for the potential influence of water loss, oil yield was further normalized to kernel dry matter. The corresponding dry-basis oil yields were 47.24 ± 0.42%, 49.61 ± 0.69%, 51.41 ± 0.57% and 51.74 ± 0.59%, respectively. Dry-matter normalization did not alter the statistical pattern among the treatments, and no significant difference was observed between the strong-aroma and sauce-aroma groups. These results indicate that increasing roasting time promoted oil release to a certain extent, but the increase in oil yield tended to level off after the strong-aroma treatment.

The increase in oil yield may be associated with roasting-induced changes in the almond kernel matrix. Thermal treatment can partially disrupt cellular structures, promote protein denaturation and reduce the resistance to oil migration during pressing, thereby facilitating oil release. Kernel moisture content is also an important factor in mechanical oil extraction because it may affect kernel plasticity, compression behavior and oil mobility during pressing. Mazaheri et al. [29] reported that thermal pretreatments markedly reduced the moisture content of Nigella sativa seeds, whereas adjustment of seed moisture before pressing substantially altered the oil extraction yield, demonstrating the importance of moisture control during mechanical oil extraction. In the present study, however, normalization to kernel dry matter caused only minor changes in the numerical oil yields and did not alter the statistical differences among the treatments. Specifically, the increase from the cold-aroma to the sauce-aroma group was 4.54 percentage points on the original-mass basis and 4.49 percentage points on the dry-matter basis. The limited effect of dry-matter normalization may be partly attributed to the relatively low initial moisture content of the almond kernels (3.04%) and the narrow moisture range among the treatments (2.70–3.04%). Thus, under the present experimental conditions, roasting-induced moisture loss contributed only marginally to the observed increase in oil yield, whereas changes in the kernel matrix and improved oil mobility were likely more important contributors. However, these findings should not be interpreted as indicating that moisture content is generally unimportant for mechanical oil extraction. A similar explanation has been reported in roasted walnut oil processing, where roasting pretreatment affected oil extraction rate and was associated with changes in physicochemical properties and oxidative stability [6]. For almond oil, Roncero et al. [5] also showed that extraction system and operating conditions could influence oil production performance, although the changes in oil composition were relatively limited. These findings support the view that processing conditions can affect oil recovery, but their effects should be interpreted together with the specific raw material and pressing conditions.

It should be noted that roasting does not necessarily lead to a continuous increase in oil yield. Álvarez-Ortí et al. [30] investigated almond oils obtained from kernels roasted at 150 °C for different times and showed that roasting mainly contributed to changes in sensory and volatile characteristics, while excessive roasting could introduce undesirable sensory notes. Based on previous studies on almond and walnut roasting, 130 °C was selected as a moderate roasting temperature in the present study. Roasting temperatures within approximately 120–150 °C have been reported to promote structural disruption, oil release, color development and volatile formation in nut materials, whereas more intensive thermal treatments may increase lipid oxidation and undesirable sensory changes [6,7,29]. Therefore, 130 °C was used to evaluate the effects of roasting time while limiting excessive thermal deterioration. In this study, the sauce-aroma group did not show a significantly higher oil yield than the strong-aroma group, indicating that further roasting offered little additional improvement in oil recovery. Nevertheless, because only one roasting temperature was investigated, the independent and interactive effects of roasting temperature and time could not be fully distinguished. Moreover, because the almond kernels had a relatively low initial moisture content and the moisture range among the roasting treatments was narrow, the influence of a broader range of kernel moisture levels on pressing performance could not be determined. Future studies should systematically evaluate different combinations of roasting temperature, roasting duration and controlled initial moisture content. Almond kernels should be equilibrated to different moisture levels before roasting and pressing, and the residual oil content of the press cake should be determined to distinguish changes in apparent oil concentration from actual improvements in mechanical extraction efficiency. Such studies would help optimize the balance among oil yield, aroma formation, nutritional quality and oxidative stability.

#### 3.1.2. Viscosity Changes

The changes in apparent viscosity of almond oils with different roasting times are shown in Figure 1B. Within the tested shear-rate range of 0.01–10 s^−1^, the viscosity of all almond oil samples showed a slight decreasing trend with increasing shear rate. Specifically, the viscosity of the cold-aroma group decreased from 42.16 to 41.05 mPa·s, corresponding to a reduction of 2.63%. The viscosity reductions in the light-aroma, strong-aroma and sauce-aroma groups were 3.82%, 3.91% and 2.69%, respectively. Overall, the magnitude of viscosity change within each treatment was relatively small, indicating that almond oil exhibited only weak shear-rate-dependent behavior under the present testing conditions and generally showed near-Newtonian flow characteristics. Among the different roasting treatments, the viscosity of almond oil generally followed the order of sauce-aroma > strong-aroma > light-aroma > cold-aroma throughout the tested range. The average viscosities of the cold-aroma, light-aroma, strong-aroma and sauce-aroma groups were 41.62, 42.38, 43.32 and 44.22 mPa·s, respectively, suggesting that increasing roasting time slightly increased the apparent viscosity of almond oil. This change may be related to mild lipid oxidation during roasting, the migration of small amounts of non-lipid minor components into the oil phase, or the presence of fine suspended particles in the oil.

The continuous phase of liquid vegetable oils is mainly composed of triacylglycerols; therefore, they usually exhibit relatively simple flow behavior. Yalcin et al. [31] reported that several vegetable oils, including hazelnut and olive oils, showed stable steady-shear behavior, and their viscosity was associated with fatty acid composition. Diamante and Lan [32] also indicated that the absolute viscosity of vegetable oils was mainly affected by oil type and temperature, and most vegetable oils behaved close to Newtonian fluids within a certain shear-rate range. In addition, Fasina and Colley [33] reported that temperature had a marked effect on vegetable oil viscosity, with viscosity decreasing notably as temperature increased. Overall, roasting time caused only slight changes in the apparent viscosity of almond oil. Although viscosity decreased slightly with increasing shear rate, the reduction was limited, suggesting only weak shear-rate-dependent behavior. Therefore, the almond oils generally maintained near-Newtonian flow characteristics, and roasting mainly resulted in a slight increase in apparent viscosity rather than a marked change in flow behavior.

#### 3.1.3. Color Characteristics

The color parameters of almond oils were affected by roasting time (Figure 1C). The L* value decreased from 25.91 ± 0.08 in the cold-aroma group to 23.61 ± 0.46 in the sauce-aroma group, indicating a gradual reduction in lightness with increasing roasting time. In contrast, the a* value increased from −0.20 ± 0.05 to 0.13 ± 0.01, showing a shift from a slightly greenish tone toward a reddish tone. The b* value increased from 1.64 ± 0.34 in the cold-aroma group to 3.65 ± 0.18 in the strong-aroma group, and remained at a relatively high level in the sauce-aroma group. These results suggest that roasting promoted the darkening of almond oil and enhanced its yellow-red color characteristics. To further describe the overall color change, ΔE and hue angle (H°) were calculated. Using the cold-aroma group as the reference, the ΔE values of the light-aroma, strong-aroma and sauce-aroma groups were 1.61, 2.51 and 3.04, respectively, indicating that the total color difference increased gradually with roasting time. Meanwhile, the H° value decreased from 97.25° in the cold-aroma group to 87.96° in the sauce-aroma group. This decrease in hue angle further indicates that the color of almond oil shifted from a greenish-yellow region toward a more yellow-red direction as roasting time increased.

The color shifts observed here may result from heat-driven reactions during roasting. During almond roasting, non-enzymatic browning, including Maillard and caramelization-related reactions, can generate brown or colored compounds. Together with lipid-soluble pigments and minor matrix-derived substances, these products may contribute to the darker yellow-red appearance of the extracted oil. Nizamlioglu and Nas [34] reported that the color parameters of roasted almonds changed significantly with roasting temperature and time, and the L* value was useful for monitoring the time of roasting. Agila and Barringer [7] also showed that roasting conditions affected both the color and volatile profile of sweet almonds, indicating that color development is closely related to thermal reactions during roasting. In addition, Lian and Chen [35] found that roasting changed the color of almonds, while shorter radio-frequency roasting caused a smaller color change than conventional oven roasting, suggesting that the extent of color change depends on the intensity and duration of heat treatment. In the present study, the decrease in L* and H° values, together with the increase in a*, b* and ΔE, indicates that increasing roasting time led to a gradual deepening of almond oil color. However, the change in b from the strong-aroma to sauce-aroma group was limited, suggesting that the yellow tone may have reached a relatively high level at the strong-aroma stage. Therefore, the color results indicate a progressive but moderate color shift during roasting, rather than a sharp change among all treatments.

#### 3.1.4. Radical-Scavenging and Reducing Capacities

The radical-scavenging and reducing capacities of almond oils under different roasting times are shown in Figure 1D,F. Overall, ABTS, DPPH and FRAP values decreased gradually with increasing roasting time. The ABTS value decreased from 1.29 ± 0.02 μmol TE/g oil in the cold-aroma group to 1.18 ± 0.04 μmol TE/g oil in the sauce-aroma group, corresponding to a reduction of 8.38%. Similarly, the DPPH value decreased from 1.37 ± 0.05 to 1.19 ± 0.04 μmol TE/g oil, with a reduction of 13.01%. The FRAP value decreased from 2.00 ± 0.02 to 1.86 ± 0.06 μmol TE/g oil, with a reduction of 6.89%. Among the three assays, DPPH showed the largest relative decrease, suggesting that the DPPH radical-scavenging capacity of almond oil may be more sensitive to roasting intensity under the present experimental conditions. Statistical analysis showed that the cold-aroma group had significantly higher ABTS and DPPH values than the strong-aroma and sauce-aroma groups (*p* < 0.05), whereas the differences between the cold-aroma and light-aroma groups were not significant. For FRAP, the sauce-aroma group was significantly lower than the cold-aroma group (*p* < 0.05), while the light-aroma and strong-aroma groups showed intermediate values. These results indicate that mild roasting caused limited changes in the antioxidant-related capacity of almond oil, whereas stronger roasting led to a measurable decrease in both radical-scavenging and reducing capacities.

The decrease in antioxidant-related capacity may be associated with the thermal sensitivity of natural antioxidant components in almond oil. Almond kernels and almond oil contain minor compounds such as tocopherols and phenolic compounds, which contribute to radical-scavenging and reducing capacities. However, these compounds may be degraded, oxidized or consumed during thermal processing, especially under stronger roasting conditions. Bolling et al. [36] reported that roasting reduced total phenols and FRAP values in almond skins, supporting the possibility that heat treatment may decrease some antioxidant-active components. In the present study, the consistent decreases in ABTS, DPPH and FRAP values suggest that the loss or consumption of heat-sensitive antioxidants may have exceeded the potential contribution of newly formed antioxidant Maillard reaction products. It should also be noted that the effect of roasting on antioxidant activity is not always consistent among nut products. Lin et al. [8] reported that phenolic components and antioxidant activities of almond kernels showed complex changes during roasting, depending on roasting temperature and duration. In roasted walnut oil, Li et al. [6] also found that roasting conditions affected ABTS, DPPH and FRAP values, indicating that antioxidant changes are closely related to roasting intensity and the specific oil matrix. Overall, increasing roasting time moderately reduced the antioxidant-related capacity of almond oil, suggesting that the potential loss of antioxidant-active components should be considered when roasting is used to improve oil yield and develop roasted flavor.

#### 3.1.5. Oxidation-Related Quality Indices

Acid value, peroxide value and TBARS value reflect lipid quality deterioration from the perspectives of free fatty acid accumulation, primary oxidation product formation and secondary oxidation product generation, respectively. As shown in Figure 1G–I, all three indices showed an overall increasing trend with increasing roasting time, suggesting that stronger roasting may promote hydrolytic and oxidative reactions in almond oil. Specifically, the acid value increased from 0.28 ± 0.04 mg KOH·g^−1^ in the cold-aroma group to 0.35 ± 0.02 mg KOH·g^−1^ in the light-aroma group, and then tended to stabilize, with values of 0.38 ± 0.02 and 0.38 ± 0.01 mg KOH·g^−1^ in the strong-aroma and sauce-aroma groups, respectively. Peroxide value and TBARS value showed similar upward trends, reaching 1.46 ± 0.08 meq O_2_·kg^−1^ and 0.46 ± 0.02 mg MDA·kg^−1^ in the sauce-aroma group, respectively, which were higher than those in the cold-aroma group. Overall, increasing roasting time increased the oxidation-related indices of almond oil, but the absolute values remained relatively low, indicating that no obvious oxidative deterioration occurred under the present processing conditions. In addition to directly inducing hydrolytic and oxidative reactions, roasting may affect oil quality through the thermal inactivation of endogenous enzymes. Lipase catalyzes the hydrolysis of storage triacylglycerols and promotes the release of free fatty acids, whereas lipoxygenase catalyzes the oxygenation of polyunsaturated fatty acids and initiates the formation of lipid hydroperoxides. Therefore, the inactivation of lipase and lipoxygenase during roasting may suppress enzymatic hydrolysis and oxidation, thereby limiting increases in acid value and peroxide value. Mazaheri et al. [29] reported that thermal pretreatment reduced lipase activity in *Nigella sativa* seeds and suggested that the inactivation of lipase and other oxidative enzymes contributed to improved oil quality and oxidative stability. Li et al. [37] also demonstrated that thermal treatment substantially inactivated lipase and lipoxygenase in lipid-rich plant materials.

The increase in acid value may be related to the release of free fatty acids during thermal processing. Roasting can disrupt cellular structures and expose oil bodies to heat and oxygen, which may facilitate hydrolysis or oxidation-related changes in lipids. Although lipase inactivation would be expected to reduce enzymatic triacylglycerol hydrolysis, the disruption of the kernel matrix may release pre-existing free fatty acids and increase the accessibility of lipids to heat, moisture and oxygen. The slight increase in acid value observed in this study may therefore reflect the combined effects of thermal disruption and partial suppression of enzymatic hydrolysis. The stabilization of acid value between the strong-aroma and sauce-aroma groups may indicate that further hydrolysis was limited, although this possibility cannot be confirmed without directly measuring residual lipase activity. Peroxide value reflects the accumulation of lipid hydroperoxides, which are primary oxidation products, while TBARS is commonly used to indicate the formation of secondary oxidation products such as malondialdehyde-like substances. Similarly, the thermal inactivation of lipoxygenase may reduce enzyme-catalyzed hydroperoxide formation. However, exposure of unsaturated lipids to heat and oxygen during roasting can simultaneously initiate non-enzymatic autoxidation. Therefore, the increases in peroxide value and TBARS observed with increasing roasting time suggest that thermally induced non-enzymatic oxidation may have exceeded the protective effect associated with lipoxygenase inactivation under the present processing conditions. Nevertheless, enzyme inactivation may have contributed to the relatively limited magnitude of these increases. Choe and Min [12] reported that edible oil oxidation is affected by temperature, oxygen, processing conditions and fatty acid composition, and that lipid hydroperoxides can further decompose into secondary products associated with quality deterioration. Therefore, the simultaneous increase in peroxide value and TBARS in the present study suggests that stronger roasting promoted early-stage lipid oxidation in almond oil. Similar changes have been reported in nut and almond oils subjected to thermal treatment or accelerated storage. Sidhu et al. [11] found that almond oils stored at 60 °C showed significant increases in peroxide value, free fatty acids, p-anisidine value and TOTOX value, indicating oxidative degradation during heating and storage. In roasted walnut oil, Li et al. [6] also reported that roasting conditions affected oxidative stability and antioxidant-related properties, further suggesting that the oxidative response of nut oils depends on the intensity of thermal treatment and the specific oil matrix.

Overall, increasing roasting time led to slight increases in acid value, peroxide value and TBARS value of almond oil. These results may represent the net outcome of two competing processes: the promotion of non-enzymatic lipid hydrolysis and oxidation by heat and oxygen, and the simultaneous suppression of enzyme-mediated reactions through lipase and lipoxygenase inactivation. Under the present roasting conditions, the former effect appeared to predominate, although the latter may have limited further deterioration. However, the relatively small absolute changes suggest that the oxidation level of almond oil remained low under the present processing conditions. Therefore, roasting time should be controlled to balance oil yield and roasted flavor formation with the maintenance of oxidative quality. Because residual lipase and lipoxygenase activities were not directly determined, their contributions to the observed changes remain inferential. Future studies should simultaneously monitor residual enzyme activities, acid value, peroxide value and secondary oxidation products during processing and subsequent storage to clarify the relationship between enzyme inactivation and almond oil stability.

### 3.2. Changes in Fatty Acid Composition

The fatty acid composition of almond oils under different roasting times is shown in Table 1. After correction of the quantitative conversion factor, fatty acid contents were expressed as mg/g oil. Oleic acid (C18:1n9c) was the predominant fatty acid in all almond oil samples, followed by linoleic acid (C18:2n6c), palmitic acid (C16:0) and stearic acid (C18:0). Oleic acid ranged from 644.37 to 647.03 mg/g oil, while linoleic acid ranged from 140.19 to 150.42 mg/g oil. Palmitic and stearic acids occurred at lower concentrations, with ranges of 43.98–44.48 mg/g oil and 14.98–15.61 mg/g oil, respectively. Oleic, palmitic and stearic acids were not significantly altered by roasting time (*p* > 0.05), whereas linoleic acid decreased from 150.42 ± 2.12 mg/g oil in the cold-aroma group to 140.19 ± 1.24 mg/g oil in the sauce-aroma group (*p* < 0.05). Similarly, SFA and MUFA showed stable levels, ranging from 60.24 to 61.12 mg/g oil and from 648.24 to 650.87 mg/g oil, respectively. In contrast, PUFA decreased from 152.30 ± 2.39 to 141.66 ± 0.98 mg/g oil with increasing roasting time. Total identified fatty acids ranged from 850.14 to 863.47 mg/g oil, which is consistent with the expected fatty acid abundance of edible oil samples. The UFA/SFA ratio remained high and showed no significant difference among treatments, indicating that the overall unsaturated fatty acid characteristics of almond oil were largely maintained.

The fatty acid profile obtained here broadly agreed with previous reports on almond oil. Most studies have reported that almond oil is characterized by high-oleic acid content, followed by linoleic acid, palmitic acid and stearic acid [2,3,4]. Roncero et al. [2] summarized that virgin almond oil is mainly composed of palmitic, oleic and linoleic acids, with oleic acid being the dominant component. Melhaoui et al. [3] also reported that the major fatty acids in sweet almond oils were oleic, linoleic and palmitic acids, although their relative levels varied among cultivars. Kodad and Socias [4] further showed that almond oil fatty acid composition can vary considerably among genotypes, especially in oleic and linoleic acids. Thus, the high-oleic profile measured in this study matches the typical composition of almond oil, although absolute levels may depend on cultivar, growing region, environmental conditions and analytical expression. The roasting-related fatty acid response observed here also partly agreed with earlier findings. Lin et al. [8] showed that roasting temperature and duration could alter almond kernel fatty acid composition and that more intense roasting may promote fatty acid breakdown, particularly in unsaturated fatty acids. In the present study, SFA and MUFA were relatively stable, whereas linoleic acid and PUFA decreased significantly as roasting time increased. This suggests that PUFA, particularly linoleic acid, were more susceptible to thermal processing than saturated and monounsaturated fatty acids under the present conditions. Nevertheless, the dominant fatty acid profile was not fundamentally changed, indicating that roasting mainly caused a moderate reduction in PUFA rather than a complete shift in almond oil fatty acid composition. Overall, almond oil maintained a high-oleic and low-saturated fatty acid profile across different roasting times. Increasing roasting time significantly reduced linoleic acid and PUFA contents, while oleic acid, SFA and MUFA remained relatively stable. These results suggest that roasting had a limited effect on the major fatty acid framework of almond oil but could reduce heat-sensitive polyunsaturated fatty acids to some extent.

### 3.3. GC-IMS Volatile Profile and Machine-Learning-Assisted Screening of Candidate Key Aroma Compounds

**Figure 2:**
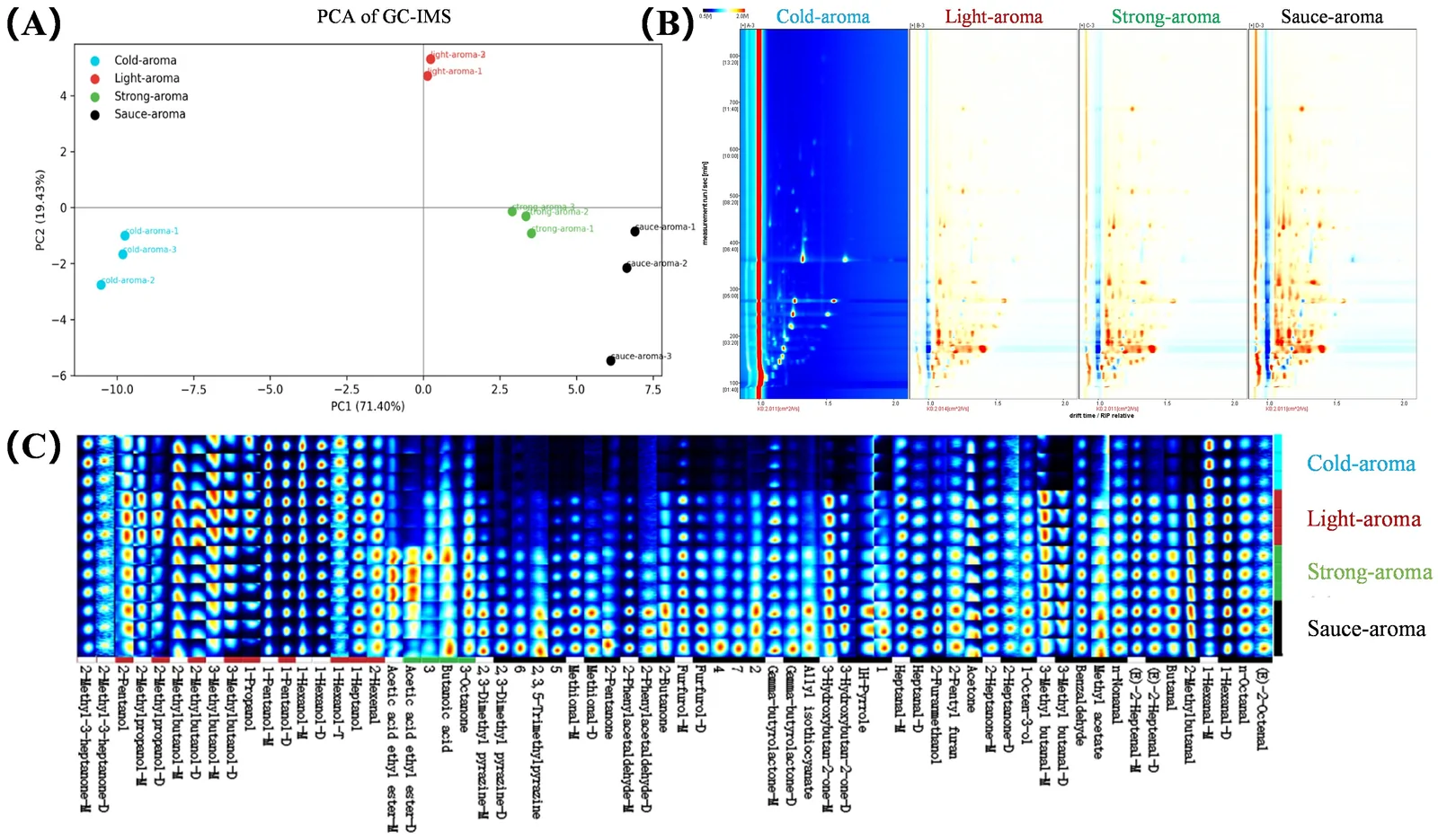
GC-IMS volatile profiles of almond oils obtained from kernels roasted at 130 °C for different times. (**A**) Principal component analysis score plot based on the Z-score-standardized peak-intensity matrix of 59 volatile signals; (**B**) the cold-aroma oil was selected as the reference and is presented as the original topographic spectrum with a blue background. The spectra of the light-aroma, strong-aroma and sauce-aroma oils were compared after subtraction of the reference spectrum. In the difference maps, white indicates a signal intensity similar to that of the reference, whereas red and blue indicate higher and lower signal intensities, respectively; (**C**) GC-IMS fingerprint profiles, in which rows represent almond oil samples, columns represent individual volatile signals, and color intensity indicates relative peak intensity. Cold-aroma, light-aroma, strong-aroma and sauce-aroma represent oils obtained from kernels roasted for 0, 5, 10 and 20 min, respectively.

GC-IMS was used to characterize the volatile profiles of almond oils with different roasting times, and 59 volatile organic compounds were retained for multivariate and machine-learning analyses (Table S3). Principal component analysis (PCA) of the Z-score-standardized GC-IMS peak-intensity matrix clearly separated the four aroma oils, with the first and second principal components (PC1 and PC2) explaining 71.40% and 14.93% of the total variance, respectively (Figure 2A). Cold-aroma oil was located on the negative side of PC1, whereas light-aroma, strong-aroma and sauce-aroma oils shifted toward the positive side. The topographic plots and fingerprint profiles further showed stronger and more numerous characteristic signals after roasting, especially for aldehydes, ketones, alcohols, furans, pyrazines and esters (Figure 2B,C). In the topographic plots, volatile signals were positioned according to their chromatographic retention time and ion-mobility drift time, while the color intensity represented the relative peak signal intensity. A stronger color therefore indicated a higher relative abundance of the corresponding volatile signal. In the fingerprint profiles, rows represented the almond oil samples and columns represented individual volatile signals; differences in color intensity reflected differences in their relative peak intensities among treatments. Quantitative comparisons and subsequent multivariate analyses were based on the peak-intensity values exported from VOCal software rather than on the visually estimated areas of the colored spots. These changes indicate that roasting shifted almond oil from a mild oil-like profile toward a stronger roasted aroma, consistent with previous reports on volatile formation in roasted almonds [9,10,37].

**Figure 3:**
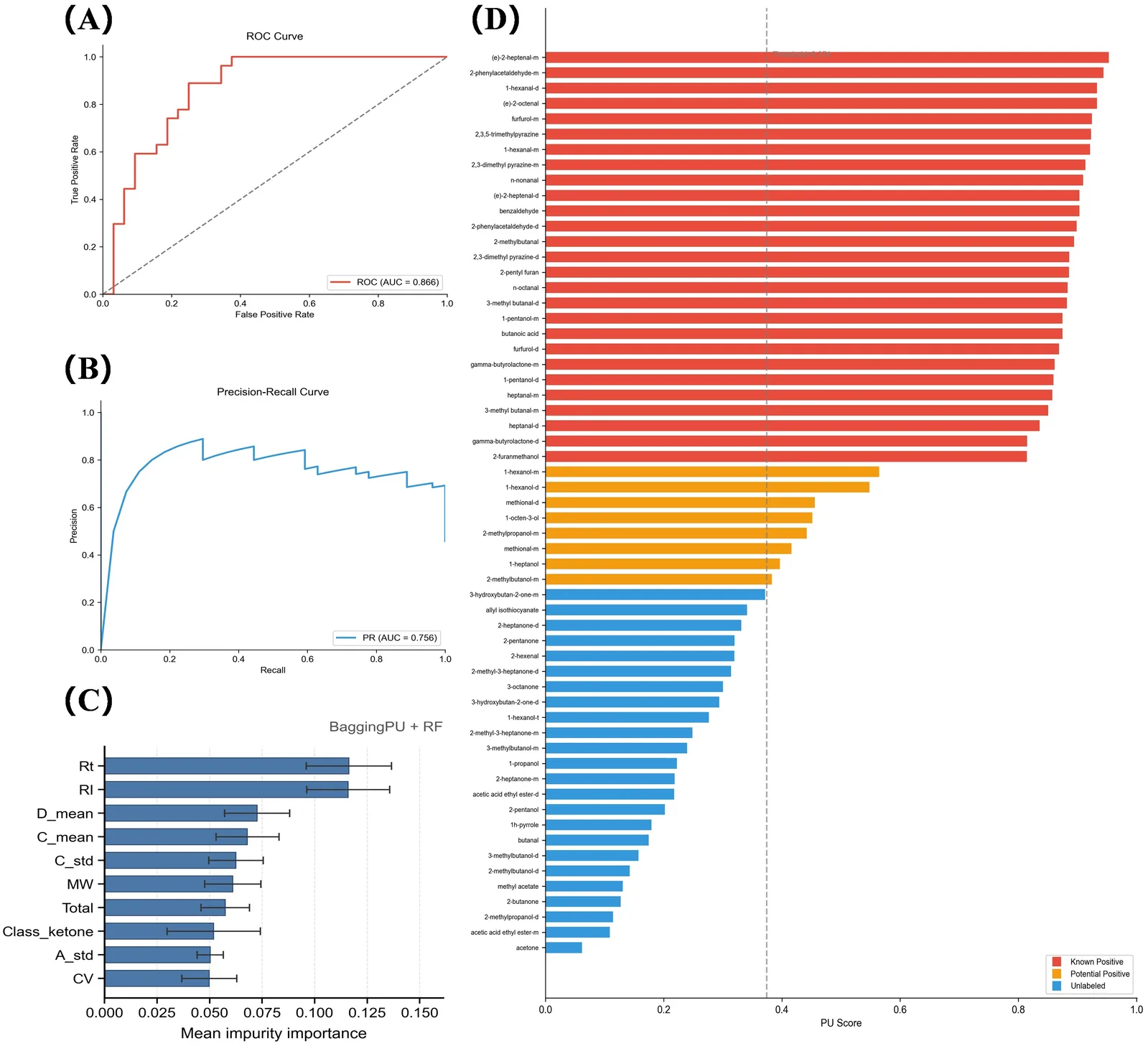
Machine-learning-assisted prioritization of candidate aroma-active compounds in almond oil. (**A**) Receiver operating characteristic curve; (**B**) precision–recall curve; (**C**) impurity-based Random Forest feature importance analysis. (**D**) PU-score ranking of the literature-supported positive and unlabeled compounds. Dotted line in Figure 3D: PU-score threshold = 0.374. ROC, receiver operating characteristic; AUC, area under the curve; PR, precision–recall; PU, positive–unlabeled; RI, retention index; Rt, retention time; CV, coefficient of variation.

A positive–unlabeled (PU) learning model was then used to screen candidate key aroma compounds from the 59 detected volatiles. The positive set contained 27 literature-supported aroma-active compounds, and the remaining 32 detected volatiles were treated as unlabeled rather than true negatives. A BaggingPuClassifier with a Random Forest base learner was evaluated by 5-fold stratified cross-validation. The model achieved a mean ROC-AUC of 0.866, a mean PR-AUC of 0.756 and a mean F1-score of 0.8617. Mean precision and recall were 0.7595 and 1.0000, respectively (Figure 3A,B). The high recall indicates that the model recovered all the literature-supported positive compounds, while the moderate precision is expected in PU learning because unlabeled samples may include hidden aroma-active compounds [15]. Random Forest feature importance analysis was used to improve model transparency [16]. Retention time and retention index were the two most influential variables, followed by D_mean, C_mean, C_std, MW and Total (Figure 3C). These results indicate that the model mainly relied on chromatographic retention behavior and roasting-dependent abundance patterns. In contrast, broad chemical-class labels showed lower importance, suggesting that the PU model did not simply classify compounds by functional group.

The PU-score ranking prioritized eight compounds from the unlabeled set as candidate aroma-active compounds above the threshold of 0.374 (Figure 3D). These candidates were 1-hexanol (monomer and dimer forms, PU_score = 0.564 and 0.548), methional (dimer and monomer forms, 0.456 and 0.416), 1-octen-3-ol (0.451), 2-methylpropanol (0.442), 1-heptanol (0.396) and 2-methylbutanol (0.383). Alcohols dominated this candidate group, whereas methional showed strong roasting-dependent accumulation, increasing from 40.80 in cold-aroma oil to 355.09 in sauce-aroma oil for the monomeric signal. By contrast, the literature-supported positive set with the highest PU_scores included aldehydes, pyrazines, furans and lactones, such as (E)-2-heptenal, 2-phenylacetaldehyde, hexanal, furfural, 2,3,5-trimethylpyrazine and gamma-butyrolactone. Overall, GC-IMS combined with PU learning separated documented aroma-active compounds from unlabeled compounds and highlighted alcohols and methional as additional candidates requiring sensory verification. These compounds should therefore be interpreted as screening candidates rather than confirmed key aroma compounds until validated by odor activity values, aroma recombination, omission tests or sensory analysis.

### 3.4. Lipidomic Changes in Almond Oil After Accelerated Oven Oxidation

**Figure 4:**
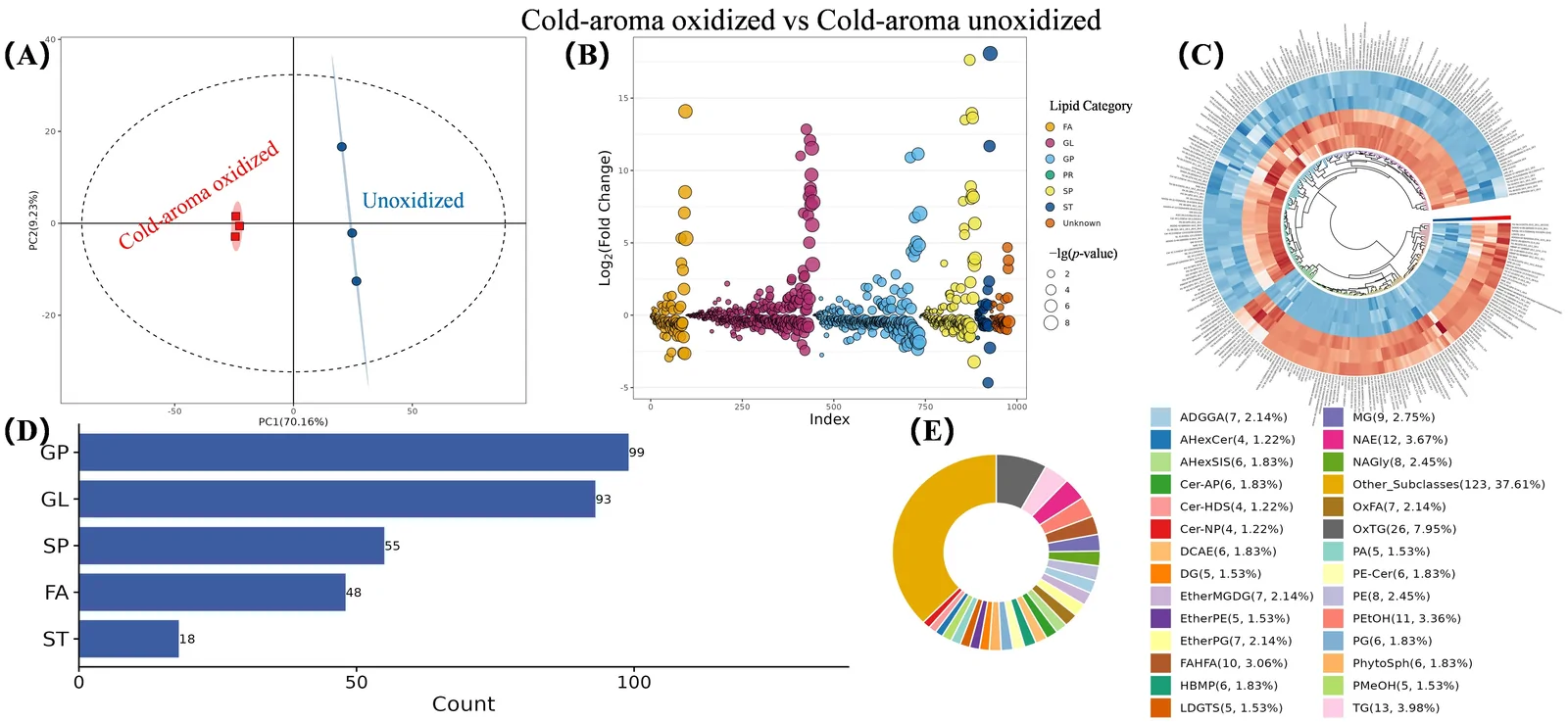
Lipidomic differences between oxidized and unoxidized cold-aroma almond oils. (**A**) Principal component analysis (PCA) score plot; (**B**) differential-lipid scatter plot, in which each point represents one lipid species, point color indicates lipid category, point size represents −log_10_(*p* value), and the log_2_ fold change was calculated as oxidized cold-aroma oil relative to unoxidized cold-aroma oil; (**C**) circular clustered heatmap of differential lipids; (**D**) distribution of differential lipids among major lipid categories, with the number beside each bar indicating the number of differential lipids; and (**E**) distribution of differential lipids among lipid subclasses. Cold-aroma oil was obtained from unroasted almond kernels, and accelerated oxidation was conducted at 60 °C for 15 days. PC1, first principal component; PC2, second principal component; FA, fatty acyls; GL, glycerolipids; GP, glycerophospholipids; PR, prenol lipids; SP, sphingolipids; ST, sterol lipids.

To further characterize lipid molecular changes after accelerated oven oxidation, lipidomic analysis was performed on cold-aroma unoxidized oil, cold-aroma oxidized oil and strong-aroma oxidized oil (Table S4). Two pairwise comparisons were conducted: cold-aroma oxidized oil vs. cold-aroma unoxidized oil, representing the effect of accelerated oxidation on cold-aroma almond oil, and strong-aroma oxidized oil vs. cold-aroma oxidized oil, representing the lipidomic difference between strong-aroma and cold-aroma almond oils after oxidation. In the comparison between cold-aroma oxidized oil and cold-aroma unoxidized oil, the PCA score plot showed a clear separation between the two groups, with PC1 and PC2 explaining 70.16% and 9.23% of the total variance, respectively (Figure 4A). This separation indicates that accelerated oven oxidation caused a marked shift in the lipid profile of cold-aroma almond oil. A total of 327 non-redundant differential lipids were identified, including 136 lipids with higher abundance in cold-aroma oxidized oil and 191 lipids with higher abundance in cold-aroma unoxidized oil (Figure 4B). Among them, 319 differential lipids had false discovery rate (FDR) values below 0.05, suggesting that the lipidomic changes induced by accelerated oxidation were extensive and statistically reliable.

At the lipid class level, the differential lipids between cold-aroma oxidized oil and cold-aroma unoxidized oil were mainly assigned to glycerophospholipids (GP, 99 lipids), glycerolipids (GL, 93 lipids), sphingolipids (SP, 55 lipids), fatty acyls (FA, 48 lipids) and sterol lipids (ST, 18 lipids) (Figure 4D). These results indicate that accelerated oxidation affected multiple lipid categories rather than a single lipid class. Among them, GP contained the largest number of differential lipids, most of which were lower in the oxidized oil, suggesting that some glycerophospholipid molecules were relatively sensitive to oxidation. GL also showed extensive changes, but this class contained both increased and decreased lipid species, indicating that oxidation caused molecular redistribution within glycerolipids rather than a uniform directional change. At the lipid subclass level, OxTG, TG, NAE, PEtOH, FAHFA, MG and PE were the major changed subclasses in the comparison between cold-aroma oxidized oil and cold-aroma unoxidized oil (Figure 4E). OxTG contained 26 differential molecules, including both increased and decreased species, indicating that accelerated oxidation led to a redistribution of oxidized triacylglycerol species rather than a simple overall increase. Previous studies have shown that triacylglycerols are the main substrates of edible oil oxidation, and thermal or photo-oxidation can generate triacylglycerol hydroperoxides and other oxidized triacylglycerol derivatives [17,18]. Redox lipidomics studies on heated vegetable oils have also demonstrated that oxidized triglycerides can be extensively annotated by mass spectrometry, supporting the usefulness of lipidomics for characterizing oil oxidation at the molecular level [19]. Thus, the pronounced OxTG changes observed here align with the typical molecular response of edible oils during oxidation. Beyond OxTG redistribution, TG molecules were generally reduced in cold-aroma oxidized oil, whereas MG molecules were consistently higher than in cold-aroma unoxidized oil. This pattern may reflect selective transformation, cleavage or depletion of some glycerolipid molecules during accelerated oxidation. Meanwhile, PE and PG subclasses were mainly decreased after oxidation, indicating that part of the glycerophospholipid fraction was reduced during the oxidation process. A similar lipidomics-based study on roasted flaxseed oil reported that roasting changed multiple lipid classes, including fatty acids, phospholipids, triacylglycerols and oxidized fatty acids, suggesting that thermal processing can reshape the lipid profile of edible oils at both class and molecular-species levels [20]. In the present study, the circular heatmap further confirmed distinct lipid-abundance patterns between cold-aroma unoxidized oil and cold-aroma oxidized oil, supporting the strong lipid remodeling effect of accelerated oxidation (Figure 4C).

**Figure 5:**
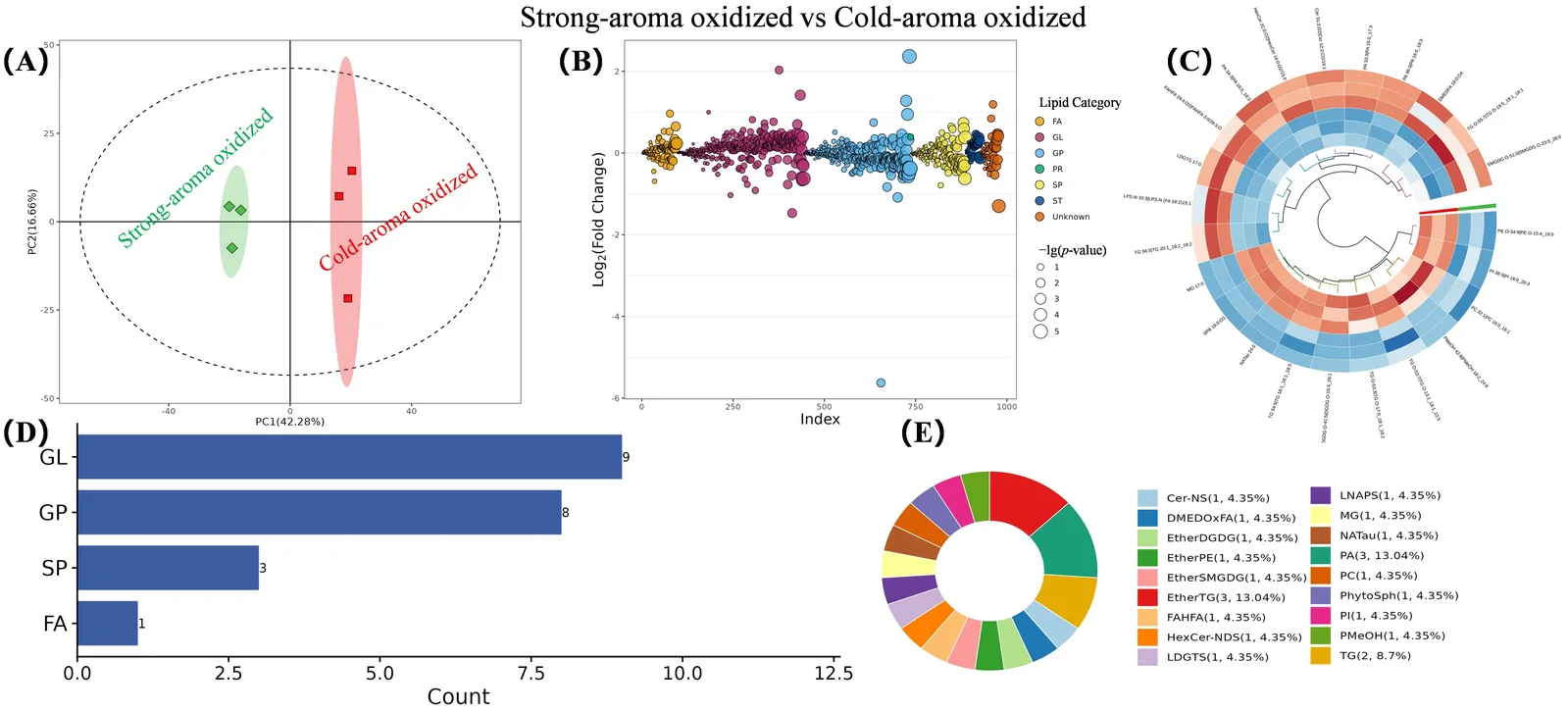
Lipidomic differences between oxidized strong-aroma and oxidized cold-aroma almond oils. (**A**) Principal component analysis (PCA) score plot; (**B**) differential-lipid scatter plot, in which each point represents one lipid species, point color indicates lipid category, point size represents −log_10_(*p* value), and the log_2_ fold change was calculated as oxidized strong-aroma oil relative to oxidized cold-aroma oil; (**C**) circular clustered heatmap of differential lipids; (**D**) distribution of differential lipids among major lipid categories, with the number beside each bar indicating the number of differential lipids; and (**E**) distribution of differential lipids among lipid subclasses. Cold-aroma and strong-aroma oils were obtained from almond kernels roasted at 130 °C for 0 and 10 min, respectively, and both oils were subjected to accelerated oxidation at 60 °C for 15 days. PC1, first principal component; PC2, second principal component; FA, fatty acyls; GL, glycerolipids; GP, glycerophospholipids; PR, prenol lipids; SP, sphingolipids; ST, sterol lipids.

Compared with the effect of accelerated oxidation, the comparison between strong-aroma oxidized oil and cold-aroma oxidized oil showed a much smaller lipidomic difference. PCA still separated strong-aroma oxidized oil from cold-aroma oxidized oil, with PC1 and PC2 explaining 42.28% and 16.66% of the total variance, respectively (Figure 5A). However, only 23 non-redundant differential lipids were detected, including 12 lipids with higher abundance in strong-aroma oxidized oil and 11 lipids with higher abundance in cold-aroma oxidized oil (Figure 5B). Among them, 12 differential lipids had FDR values below 0.05. These results indicate that roasting time affected the lipid profile after oxidation, but the magnitude of this effect was much smaller than that caused by accelerated oxidation itself. The 23 differential lipids between strong-aroma oxidized oil and cold-aroma oxidized oil were mainly distributed in GL (nine lipids), GP (eight lipids), SP (three lipids) and FA (one lipid) (Figure 5D). At the subclass level, PA was the most notable subclass, with all three PA molecules showing higher abundance in strong-aroma oxidized oil (Figure 5E). Specifically, PA 36:3|PA 18:0_18:3, PA 34:3|PA 16:0_18:3 and PA 33:3|PA 16:0_17:3 were increased in strong-aroma oxidized oil, with fold changes of 5.16, 2.43 and 1.93, respectively. This suggests that strong-aroma roasting may influence the subsequent oxidation-related remodeling of phospholipid-derived molecules. In contrast, several molecules, including MG 17:0, TG 54:5|TG 18:1_18:1_18:3, PE O-34:9, DGDG O-41:5, SPB 18:0; O3 and NATau 24:0, were lower in strong-aroma oxidized oil than in cold-aroma oxidized oil. Therefore, the difference between strong-aroma and cold-aroma oxidized oils was characterized by selective changes in specific lipid molecules rather than a broad shift in all lipid classes. Because lipidomic analysis was conducted only on selected oxidation-related comparisons, the interaction between roasting time and oxidation should be further verified using all roasting groups before and after oxidation.

Overall, accelerated oven oxidation substantially remodeled the almond oil lipidome, mainly involving glycerophospholipids, glycerolipids, sphingolipids and fatty acyls. The major molecular changes included redistribution of OxTG species, reduction in several TG and glycerophospholipid molecules, and accumulation of MG and selected sphingolipid-related molecules. In contrast, the difference between strong-aroma oxidized oil and cold-aroma oxidized oil was more limited and mainly reflected selective changes in PA, TG, MG, EtherPE, DGDG and sphingolipid-related molecules. These findings indicate that accelerated oxidation was the dominant factor driving lipidomic variation, while roasting time further modulated the molecular response of almond oil after oxidation. Future studies should include all roasting treatments before and after oxidation and perform an integrated PCA to distinguish the effects of roasting time, oxidation and their interaction. Because lipidomic analysis was limited to selected treatments, the interaction between roasting time and oxidation should be further evaluated using all roasting groups before and after oxidation. In addition, GC-IMS analysis of the same oil samples before and after accelerated oxidation could provide complementary information on oxidation-derived volatile markers, including aldehydes, ketones and alcohols. However, the GC-IMS and lipidomic analyses in the present study were based on different sample designs; therefore, direct correlations between volatile markers and differential lipids were not performed. Future studies should apply both techniques to the same sample set to clarify the relationships between lipid degradation and volatile formation.

## 4. Conclusions

This study showed that roasting time markedly affected almond oil yield, quality characteristics, aroma formation and oxidative lipid remodeling. Increasing roasting time improved oil yield, but the increase tended to level off at higher roasting intensity. Stronger roasting promoted color deepening and volatile aroma formation, while also causing a moderate decrease in antioxidant-related capacity and slight increases in oxidation-related indices. Although almond oil maintained a high-oleic fatty acid profile, linoleic acid and total PUFA contents decreased with increasing roasting intensity. GC-IMS combined with PU learning identified eight unlabeled volatiles, mainly alcohols and methional, as additional aroma candidates, while known positives included aldehydes, pyrazines, furans and lactones. Lipidomics further revealed that accelerated oxidation mainly involved glycerophospholipids, glycerolipids, sphingolipids and fatty acyls. Overall, roasting time should be carefully controlled to balance oil recovery, aroma development and oxidative quality. Further sensory validation, aroma recombination or omission experiments, and lipidomic analysis covering all roasting times before and after oxidation are needed to clarify aroma contribution and oxidation mechanisms. These findings provide a basis for optimizing almond oil roasting and pressing processes.

## Figures and Tables

**Table 1:** Fatty acid composition and related indices of almond oils with different roasting times.

Fatty Acid/Index	Cold-Aroma	Light-Aroma	Strong-Aroma	Sauce-Aroma
C14:0	1.16 ± 0.33 a	1.25 ± 0.33 a	1.03 ± 0.28 a	1.19 ± 0.20 a
C16:0	44.14 ± 3.30 a	44.04 ± 2.62 a	44.48 ± 3.65 a	43.98 ± 3.95 a
C16:1	3.12 ± 0.47 a	3.15 ± 0.47 a	3.15 ± 0.47 a	3.22 ± 0.47 a
C17:1	0.72 ± 0.14 a	0.69 ± 0.14 a	0.66 ± 0.16 a	0.66 ± 0.25 a
C18:0	15.02 ± 1.08 a	14.98 ± 0.81 a	15.61 ± 0.30 a	15.08 ± 1.08 a
C18:1n9c	647.03 ± 2.32 a	645.28 ± 3.67 a	645.44 ± 4.10 a	644.37 ± 0.84 a
C18:2n6c	150.42 ± 2.12 a	145.34 ± 0.38 b	141.90 ± 2.24 bc	140.19 ± 1.24 c
C18:3n6	1.00 ± 0.14 a	0.84 ± 0.09 a	0.84 ± 0.16 a	0.78 ± 0.20 a
C20:4n6	0.88 ± 0.14 a	0.78 ± 0.06 a	0.75 ± 0.16 a	0.69 ± 0.20 a
SFA	60.31 ± 3.28 a	60.28 ± 2.49 a	61.12 ± 3.46 a	60.24 ± 4.14 a
MUFA	650.87 ± 2.42 a	649.12 ± 3.94 a	649.24 ± 4.40 a	648.24 ± 1.18 a
PUFA	152.30 ± 2.39 a	146.96 ± 0.39 b	143.50 ± 1.96 bc	141.66 ± 0.98 c
UFA	803.16 ± 3.06 a	796.08 ± 3.78 ab	792.74 ± 4.04 b	789.90 ± 2.14 b
Total identified FA	863.47 ± 1.88 a	856.36 ± 1.30 ab	853.86 ± 1.44 b	850.14 ± 6.11 b
UFA/SFA	13.35 ± 0.77 a	13.22 ± 0.61 a	13.00 ± 0.83 a	13.15 ± 0.90 a

Values are expressed as the mean ± standard deviation of three independent replicates. Except for the UFA/SFA ratio, fatty acid contents and summed indices are expressed as mg/g oil. Different lowercase letters within the same row indicate significant differences among the four roasting treatments (*p* < 0.05); values sharing the same letter are not significantly different. Cold-aroma, light-aroma, strong-aroma and sauce-aroma represent oils obtained from kernels roasted at 130 °C for 0, 5, 10 and 20 min, respectively. FA, fatty acids; SFA, saturated fatty acids; MUFA, monounsaturated fatty acids; PUFA, polyunsaturated fatty acids; UFA, unsaturated fatty acids; UFA/SFA, ratio of unsaturated to saturated fatty acids.

## Data Availability

The raw data supporting the conclusions of this article will be made available by the authors on request.

## Supplementary Materials

The following supporting information can be downloaded at: https://www.mdpi.com/article/10.3390/foods15162842/s1, Table S1: Information on the 20 Published Datasets Used to Construct the Litera-ture-Supported Positive Class. Table S2: Model performance, signal-level prediction results, and impurity-based fea-ture-importance ranking of the PU-learning model. Table S3: Peak-intensity data for the 59 volatile signals detected by GC-IMS in almond oils ob-tained from kernels roasted for different times. Table S4. Lipid-abundance matrix for all samples included in the lipidomic analysis.
